# EZH2 presents a therapeutic target for neuroendocrine tumors of the small intestine

**DOI:** 10.1038/s41598-021-02181-7

**Published:** 2021-11-23

**Authors:** Elham Barazeghi, Per Hellman, Olov Norlén, Gunnar Westin, Peter Stålberg

**Affiliations:** Department of Surgical Sciences, Uppsala University, Uppsala University Hospital, Rudbeck Laboratory, 751 85 Uppsala, Sweden

**Keywords:** Cancer, Endocrinology

## Abstract

Small intestinal neuroendocrine tumors (SI-NETs) are slow-growing tumors that seem genetically quite stable without highly recurrent mutations, but are epigenetically dysregulated. In contrast to the undetectable expression of the enhancer of zeste homolog 2 (EZH2) histone methyltransferase in the enterochromaffin cells of the small intestine, we found high and differential expression of EZH2 in primary SI-NETs and corresponding metastases. Silencing EZH2 in the SI-NET cell line CNDT2.5 reduced cell proliferation and induced apoptosis. Furthermore, EZH2 knockout inhibited tumor progression in a CNDT2.5 SI-NET xenograft mouse model, and treatment of SI-NET cell lines CNDT2.5 and GOT1 with the EZH2-specific inhibitor CPI-1205 decreased cell viability and promoted apoptosis. Moreover, CPI-1205 treatment reduced migration capacity of CNDT2.5 cells. The EZH2 inhibitor GSK126 also repressed proliferation of CNDT2.5 cells. Recently, metformin has received wide attention as a therapeutic option in diverse cancers. In CNDT2.5 and GOT1 cells, metformin suppressed EZH2 expression, and inhibited cell proliferation. Exposure of GOT1 three-dimensional cell spheroids to CPI-1205 or metformin arrested cell proliferation and decreased spheroid size. These novel findings support a possible role of EZH2 as a candidate oncogene in SI-NETs, and suggest that CPI-1205 and metformin should be further evaluated as therapeutic options for patients with SI-NETs.

## Introduction

Small intestinal neuroendocrine tumors (SI-NETs) arise from enterochromaffin cells in the gastrointestinal tract with annual incidence of 1–3 per 100,000. These tumors are slow growing (Ki-67 proliferation index is often < 2%), but most patients are diagnosed at a late stage with distant metastases, most commonly in the liver, and with a 5-year survival rate around 60%^1,2^. SI-NETs can produce excess hormones, which can give rise to carcinoid syndrome (flushing and diarrhea) in approximately 20% of patients^3^. Many of the patients also present with local tumor-related symptoms such as abdominal pain and acute bowel obstruction. The main therapeutic option for patients at earlier stages of the disease is curative surgery by resection of the primary tumor. However, surgery has no survival advantage for patients who have a metastatic disease but no local tumor-related symptoms^4^. Therefore, identifying new potential therapeutic targets is needed to implement personalized treatment, and to improve prognosis.

Previous studies have shown that SI-NETs seem genetically quite stable with no highly recurrent mutations^5^, but are epigenetically dysregulated^6–8^. Loss of one copy of chromosome 18 is the most common genetic aberration in these tumors (60–90%)^9,10^, while pathogenic mutations of CDKN1B (encoding p27) have been found in a small subset of tumors (8%)^11,12^. However, the molecular features of SI-NETs are not yet fully understood.

The enhancer of zeste homolog 2 (EZH2) is a histone methyltransferase that catalyzes trimethylation of histone H3 lysine 27 (H3K27me3), and works as an essential component of the polycomb repressive complex 2 (PRC2). H3K27me3, as an epigenetic marker, reprograms epigenetic landscape and gene expression, and is thereby associated with various pathways involved in tumorigenesis. EZH2 functions as a master epigenetic regulator of cell cycle progression and cell lineage determination, and is therefore related to many diseases, including cancer^13,14^. Defects in EZH2 have been described in multiple cancer types, and its overexpression is associated with a high proliferation rate, aggressive tumor progression and poor survival^15–18^. However, how EZH2 functions in SI-NETs is unknown.

The important role of EZH2 in many types of cancer has triggered interest in therapies targeting EZH2, and several small molecule inhibitors of EZH2 have recently been developed. Although most of these compounds are still in preclinical development, some of the agents, such as CPI-1205, have moved into phase I/II clinical trials^19^. CPI-1205 is an orally bioavailable compound that binds to the EZH2 catalytic domain and partially overlaps with the S-adenosylmethionine (SAM) binding site^20^. CPI-1205 was evaluated in a phase I clinical trial in patients with B-cell lymphoma (NCT02395601). Furthermore, CPI-1205 is currently being evaluated in a phase I/II clinical trial for advanced solid tumors (NCT03525795), and a phase I/II clinical trial for metastatic castration-resistant prostate cancer (NCT03480646)^21^. GSK126, another highly selective and SAM-competitive inhibitor of EZH2^22^ showed modest anticancer activity in phase I clinical trial in patients with advanced hematologic and solid tumors (NCT02082977)^23^. However, in a recent mouse study, GSK126 treatment resulted in suppression of antitumor immunity, and therefore, modulation of the tumor immune microenvironment could enhance the activity of GSK126^24^.

Metformin, the most widely used anti-diabetic drug, is emerging as a potential anti-cancer agent according to different epidemiological studies and meta-analyses^25,26^, although the molecular mechanisms of action remain unresolved. The direct anti-tumor effects of metformin appear to be modulated through activation of the adenosine monophosphate-activated protein kinase (AMPK) and inhibition of the mammalian target of rapamycin (mTOR) signaling pathway, which leads to inhibition of cell growth and proliferation^27^. In ovarian cancer cells, the anti-tumor effect of metformin has been reported to be mediated through reduced expression of EZH2 and suppressed level of H3K27me3^28^. Metformin is being evaluated in clinical trials in various types of cancers, and the combination of metformin with chemotherapy has shown promising results in several clinical trials^29^.

In this study, we investigated the expression level of EZH2 and found a possible role for EZH2 as a candidate oncogene in SI-NET cells in vitro and in a xenograft mouse model. Moreover, we revealed that inhibition of EZH2 using the clinical trial drug CPI-1205 or metformin inhibited SI-NET tumor growth in vitro.

## Results

### EZH2 was differentially expressed in SI-NETs

**Figure 1:**
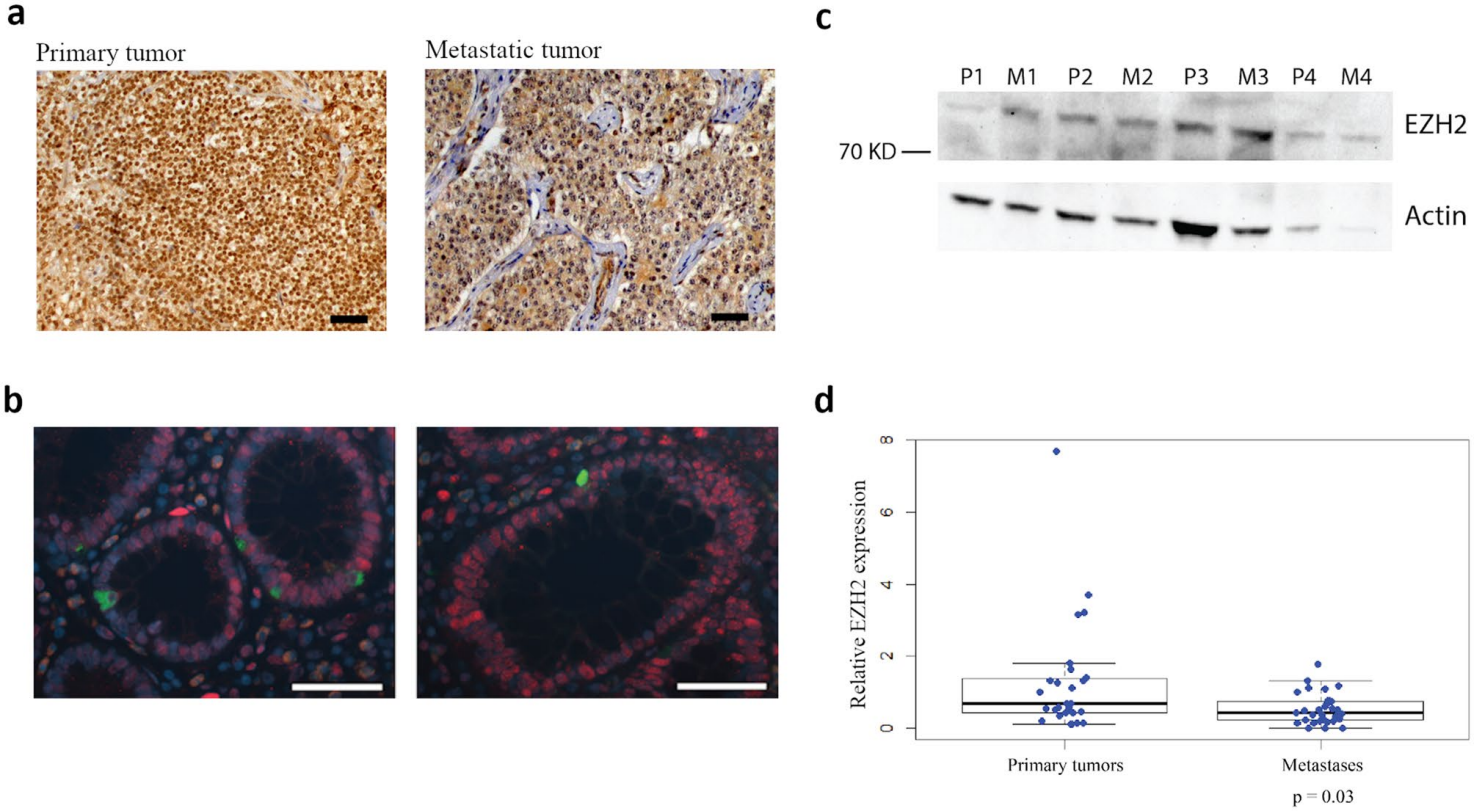
EZH2 expression in SI-NETs. (**a**) Representative results from immunohistochemical analysis of EZH2 in 17 PTs and 21 paired MTs; positive staining regardless of strength in a primary and a metastatic tumor. Scale bar 50 µm. (**b**) Immunofluorescent double staining for chromogranin A and EZH2. Chromogranin A-positive cells (green) are negatively stained for EZH2 (red) in normal small intestinal tissue. Scale bar 50 µm. (**c**) Western blot analysis of EZH2 in 4 PTs and paired MTs (n = 8). Actin was detected on the same membrane as loading control, and full-length blots are presented in Supplementary Figure S1. (**d**) mRNA expression level of EZH2 in PTs (n = 27) and paired MTs (n = 33). Wilcoxon–Mann–Whitney *U* test result displayed in the boxplot (*p* = 0.03).

To investigate expression of EZH2 in SI-NETs, immunohistochemical analysis was performed in 17 primary tumors (PT) and 21 paired metastases (MT) (Fig. 1a). All tumors stained positively for EZH2, regardless of strength. In contrast, chromogranin A-positive cells in the normal small intestinal tissue stained negatively for EZH2 (Fig. 1b). These cells represent the enterochromaffin cell of origin of SI-NETs. We also performed western blot analysis for 4 PTs and paired MTs, and found variable expression levels of EZH2 in the eight analyzed tumors (Fig. 1c). EZH2 mRNA expression analysis also revealed variable levels of expression in 60 SI-NETs, but slightly reduced expression of EZH2 in MTs (n = 33) compared to the paired PTs (n = 27) (Fig. 1d). Therefore, the results demonstrate that EZH2 is highly differentially expressed in SI-NETs.

### EZH2 knockout inhibited tumor growth in SI-NETs in vitro and in vivo

**Figure 2:**
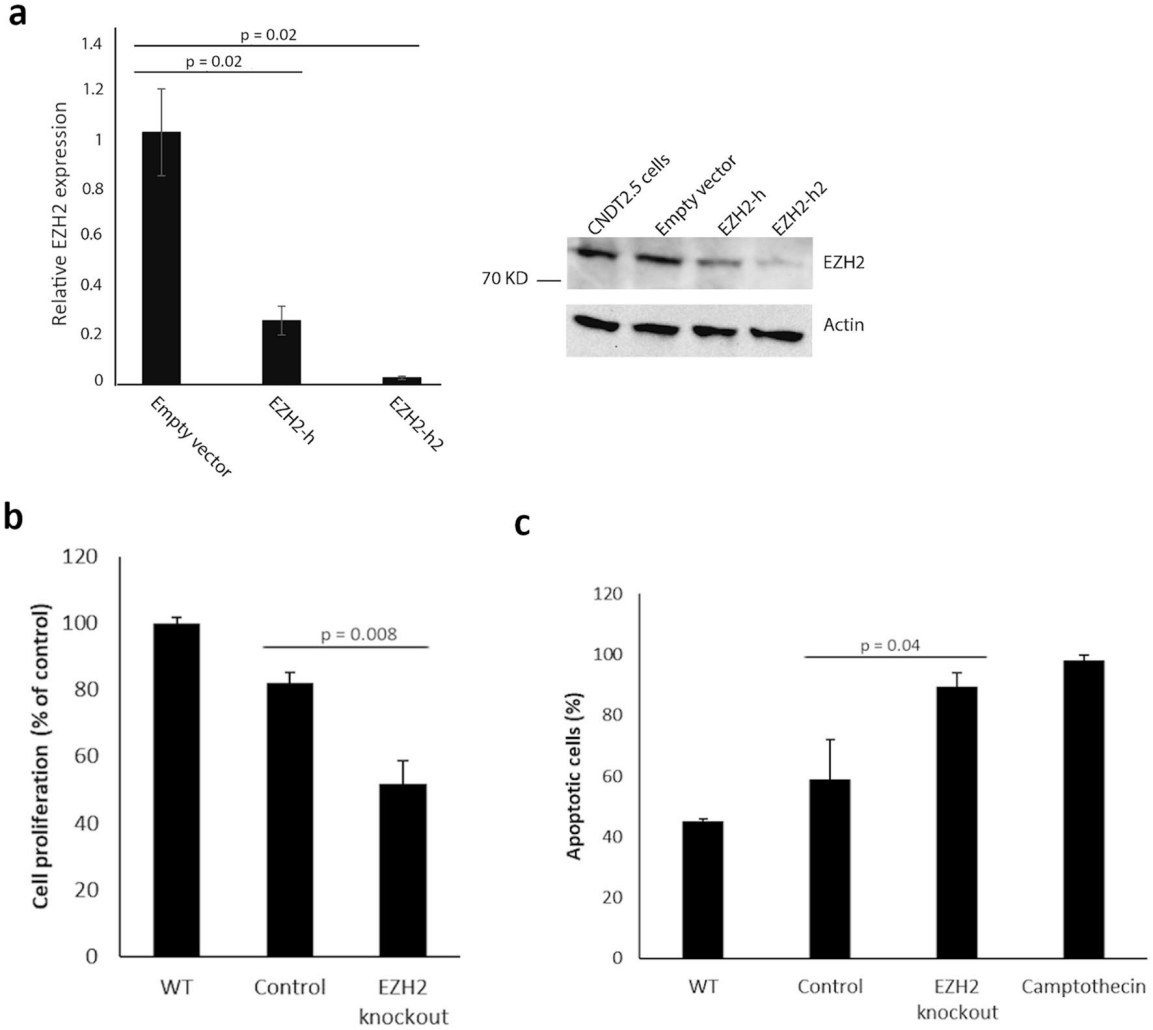
EZH2 knockout in SI-NET cells. (**a**) CNDT2.5 cells were transfected with CRISPR double nickase plasmids (EZH2-h or EZH2-h2) or control double nickase plasmid (empty vector), and were selected for puromycin resistance. Efficient knockout of EZH2 was obtained at mRNA and protein level (full-length blots are found in Supplementary Figure S1). EZH2-h2 plasmids were used for further experiments. (**b**) Cell viability measured for 24 h, seventy-two hours after transfection under 0.5 µg/mL puromycin selection. (**c**) Apoptosis was analyzed by quantifying cytoplasmic histone-associated-DNA-fragments, 72 h after transfection. Incubation in 0.1 μg/mL camptothecin was used as a positive control. Data shown are means ± SD of triplicate.

To explore the potential role of EZH2 in small intestinal neuroendocrine tumorigenesis, we used the SI-NET cell line CNDT2.5. EZH2 mRNA and protein expression was efficiently knocked out by transfection of EZH2 CRISPR double nickase plasmids using two different plasmid-constructs (EZH2-h and -h2) (Fig. 2a). The expression of EZH2 was nearly abolished by EZH2-h2 transfection, which was used in further experiments. Knockout of EZH2 expression significantly reduced cell proliferation rate (p = 0.008) and induced apoptosis (p = 0.04) in EZH2 knockout cells compared to the empty vector-transfected (control) and wild type (WT) cells (Fig. 2b,c). These results suggested an important role for EZH2 in SI-NET tumorigenesis.

**Figure 3:**
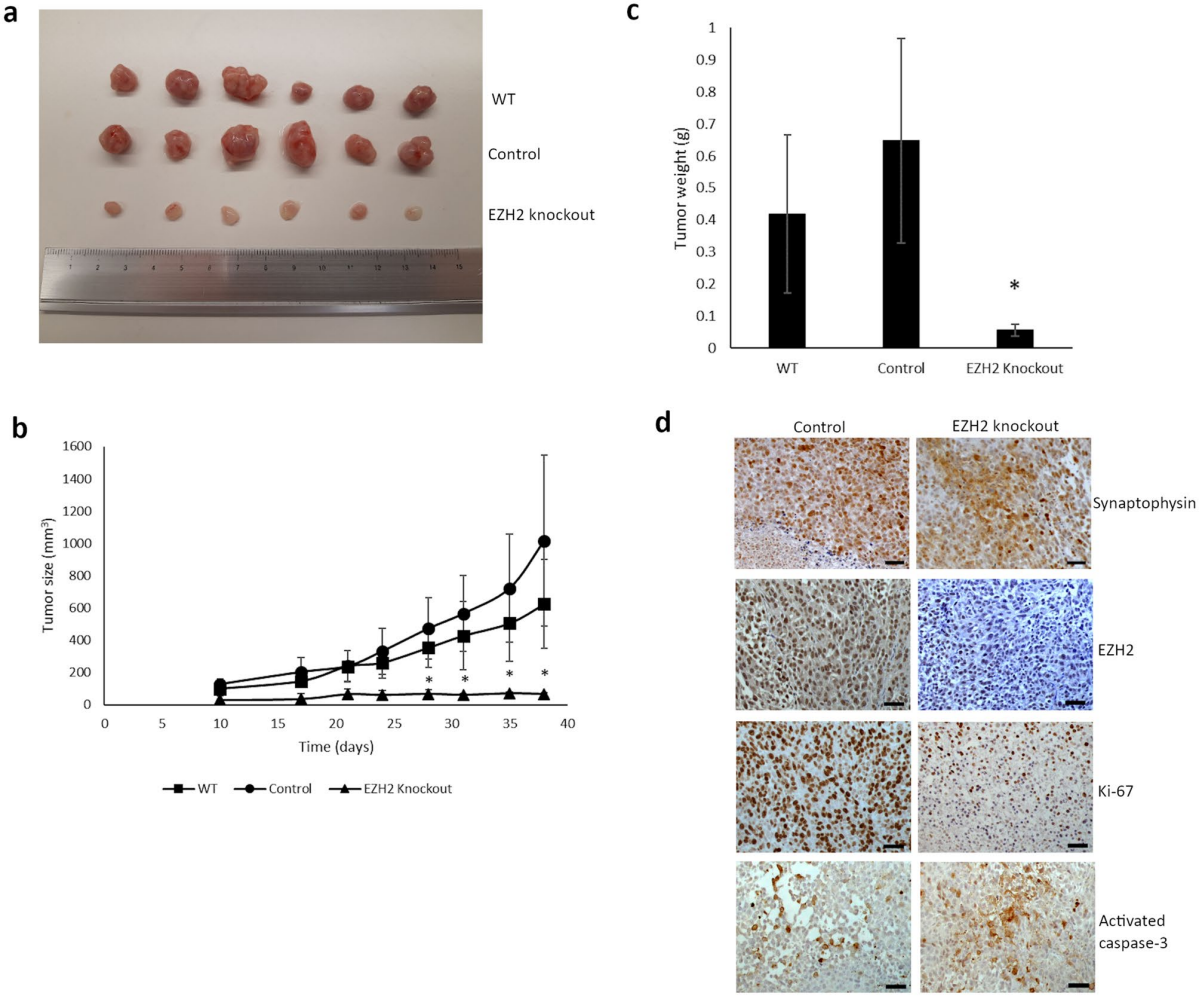
EZH2 knockout in vivo. (**a**) NMRI-nude female mice (n = 18) were randomly distributed into three groups, and CNDT2.5 WT, control or EZH2 knockout cells were subcutaneously inoculated in the hind flank of the mice. The tumors were dissected after 38 days. (**b**) Tumor volume was monitored by caliper measurements. Six mice per group were used, and the data represent means ± SD. (**c**) Xenograft tumor weight is presented as means ± SD. Tumor volumes and weights in the three groups were compared using one-way analysis of variance, and Bonferroni correction was used to adjust the *p* values (*, p < 0.001). (**d**) Representative results from immunohistochemical analysis of synaptophysin, EZH2, Ki-67, and activated caspase-3 in tumors dissected from the control and the EZH2 knockout groups. Scale bar 50 µm.

To further evaluate whether EZH2 is involved in tumorigenicity of SI-NETs in vivo, we used a tumor xenograft mouse model. 5 × 10^6^ WT, control or EZH2 knockout CNDT2.5 cells were implanted subcutaneously on the right hind flank of mice, and were allowed to grow and form tumors for 38 days (Fig. 3a). The EZH2 knockout group showed a significant suppression of tumor growth compared to the control and WT groups (p < 0.001) (Fig. 3b). Consistently, the tumor weight in the EZH2 knockout group was lower than in the control and WT groups (Fig. 3c). Immunohistochemical analysis of the neuroendocrine cell marker synaptophysin and EZH2 was performed in the dissected tumors. While EZH2 was expressed in the control group, the EZH2 knockout group displayed negative staining. All tumors stained positive for synaptophysin (Fig. 3d). To evaluate proliferation rate and apoptosis in the dissected tumors, we analyzed expression of Ki-67 and activated caspase-3 by immunohistochemistry. Tumors in the control group showed higher proliferation rate as almost all cell nuclei stained positive for Ki-67, whereas the EZH2 knockout group displayed a mixture of positive and negative cells. The immunohistochemistry of the activated caspase-3 showed higher proportion of apoptotic cells in the EZH2 knockout group than the control group. Representative staining results are shown in Fig. 3d. Taken together, these results demonstrate that silencing EZH2 reduces tumorigenicity of SI-NET cells.

### EZH2 inhibition reduced viability and induced apoptosis in SI-NET cells

**Figure 4:**
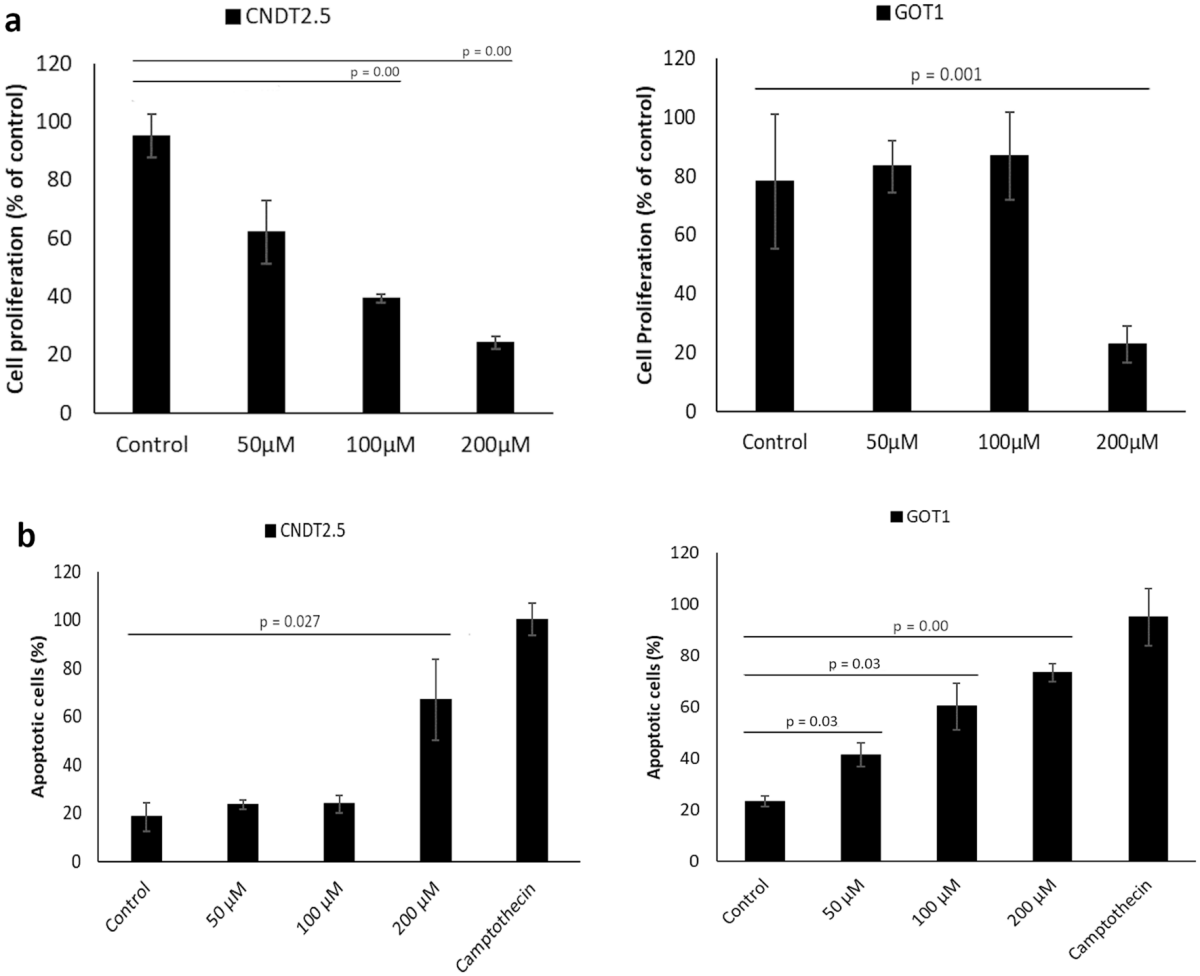
CPI-1205 treatment reduced proliferation and induced apoptosis in SI-NET cells. (**a**) Cell proliferation was determined in CNDT2.5 and GOT1 cells after treatment with CPI-1205 for 72 and 48 h, respectively. (**b**) Treatment effect on apoptosis was analyzed by quantifying cytoplasmic histone-associated-DNA-fragments under the same conditions. Incubation with 0.1 μg/mL camptothecin for 48 h was used as a positive control. Data shown are means ± SD of triplicate.

To investigate a possible clinical application of EZH2 inhibition in SI-NETs, we evaluated whether the small molecule inhibitors of EZH2 can effectively inhibit the proliferation of SI-NET cells. Treatment of CNDT2.5 cells with the selective EZH2 inhibitors EPZ-6438 and GSK126 for 3 days was insufficient to induce substantial reduction in cell viability. After 6 days of treatment, GSK126 significantly reduced proliferation of CNDT2.5 cells (Supplementary Figure S2), whereas EPZ-6438 showed no effect (data not shown). This could be explained by the EPZ-6438 having a pentacyclic molecular structure, and therefore a more limited penetration into the cells^30^. We also evaluated the effect of CPI-1205, an EZH2 specific inhibitor which is currently being tested in clinical trials. The SI-NET cell lines, CNDT2.5 and GOT1, were treated with different concentrations of CPI-1205 for 72 and 48 h, respectively, and cell proliferation and apoptosis were assessed. CPI-1205 treatment significantly reduced the proliferation (Fig. 4a) and induced apoptosis (Fig. 4b) of both cell lines compared to the control cells. Apoptosis was determined by quantifying cytoplasmic histone-associated-DNA-fragments. CPI-1205 treatment showed no effect on the proliferation and apoptosis of the CNDT2.5 EZH2 knockout cells, confirming the selectivity of the inhibitor for EZH2 (Supplementary Figure S3a).

### Metformin decreased EZH2 expression and inhibited SI-NET cell proliferation

**Figure 5:**
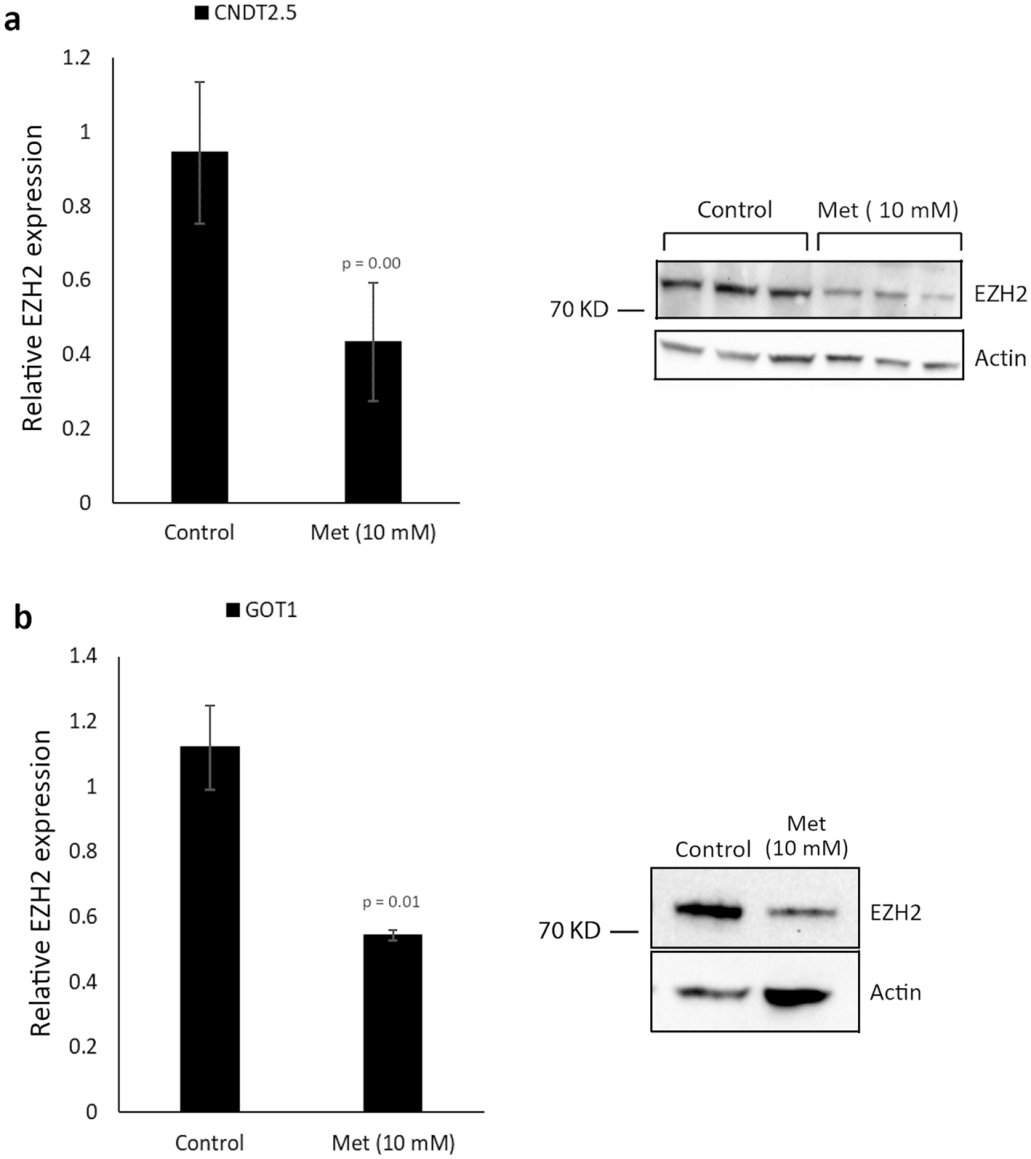
Metformin treatment reduced expression of EZH2 in SI-NET cells. (**a**) Reduced expression of EZH2 in CNDT2.5 cells after 48 h. Western blot analysis is presented for triplicates. (**b**) Reduced EZH2 expression in GOT1 cells after 72 h. Western blot analysis is presented for a pooled lysate of triplicates. Full-length blots are found in Supplementary Figure S1. Data presented are means ± SD of triplicate.

Next, we examined whether metformin reduces expression of EZH2 in SI-NETs, as has previously been demonstrated in ovarian cancer^28^. Therefore, CNDT2.5 and GOT1 cells were treated with metformin for 48 and 72 h, respectively, and the expression of EZH2 was assessed. Q-PCR and western blot analysis demonstrated that metformin suppressed EZH2 mRNA and protein expression in both cell lines (Fig. 5a,b).

**Figure 6:**
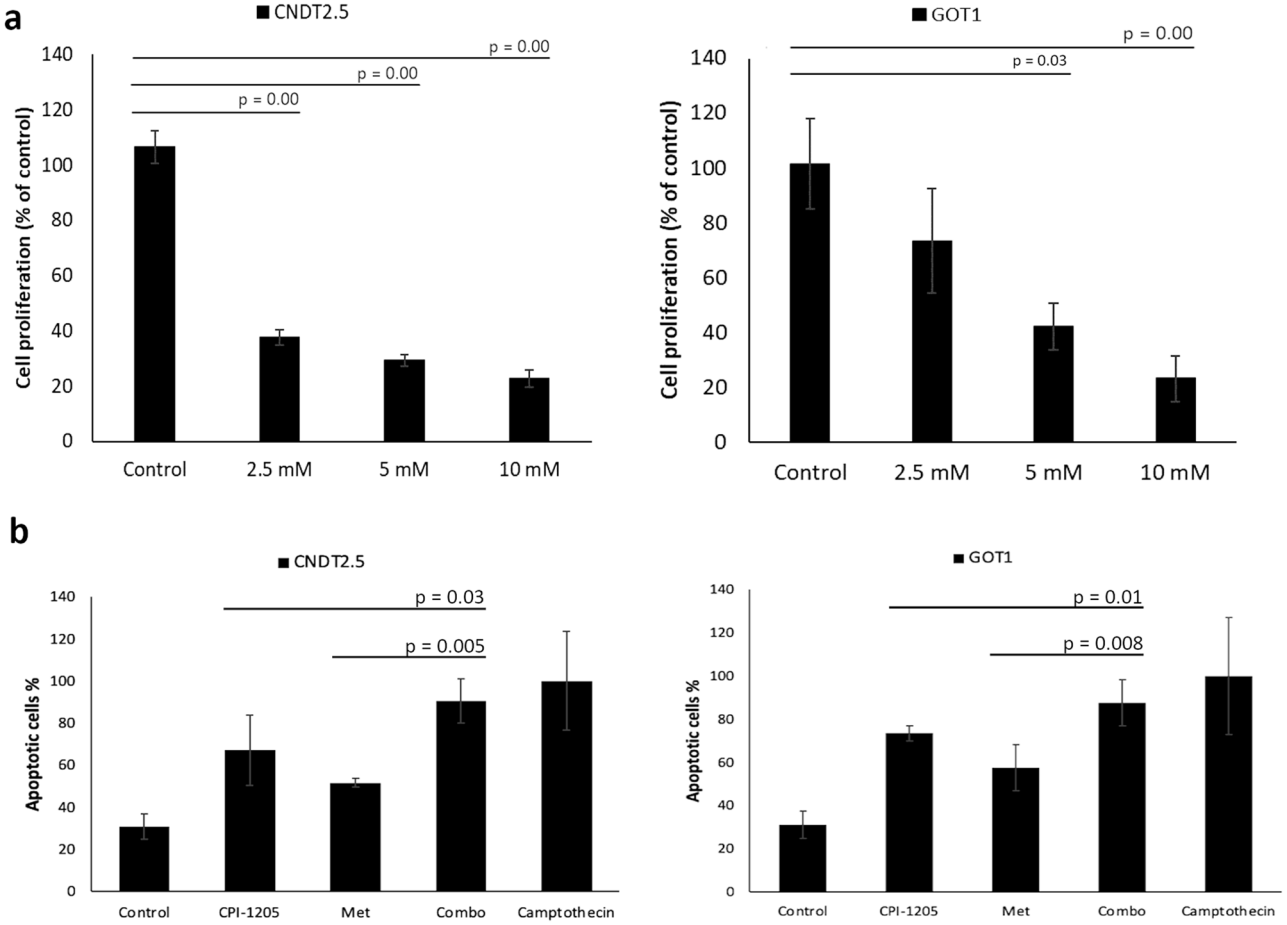
Metformin treatment reduced proliferation and, in combination with CPI-1205, induced apoptosis in SI-NET cells. (**a**) Cell proliferation was determined in CNDT2.5 and GOT1 cells after 48 and 72 h, respectively. (**b**) CNDT2.5 and GOT1 cells were treated with CPI-1205 (200 μM), metformin (Met) (10 mM), or a combination of both (Combo), followed by apoptosis assay. Data shown are means ± SD of triplicate.

Given that metformin suppressed expression of EZH2 in SI-NET cells, the effect on SI-NET tumor growth was investigated. CNDT2.5 and GOT1 cells were treated with increasing concentrations of metformin (2.5, 5 and 10 mM) for 48 and 72 h, respectively, and cell proliferation and apoptosis were assessed. Metformin treatment significantly reduced the proliferation of SI-NET cells compared to the control cells (Fig. 6a), but there was no effect on apoptosis (data not shown). Moreover, the effect of metformin treatment was assessed in CNDT2.5 EZH2 knockout cells. We found that although metformin treatment reduced cell proliferation in the knockout cells, the treatment had a stronger inhibition effect in WT cells with high level of EZH2 expression, whereas no effect was detected in the induction of apoptosis (Supplementary Figure S3b).

Next, to investigate how the combination of CPI-1205 and metformin affects the growth of SI-NET cells, CNDT2.5 and GOT1 cells were treated with the two drugs. Compared with the treatment of CPI-1205 or metformin alone, induction of apoptosis increased significantly upon treatment with combination of the two drugs in both cell lines (Fig. 6b). In contrast, the effect of the combined treatment on cell proliferation was not significantly different from the effects of mono-treatments (Supplementary Figure S4).

### CPI-1205 treatment inhibited migration of CNDT2.5 cells

**Figure 7:**
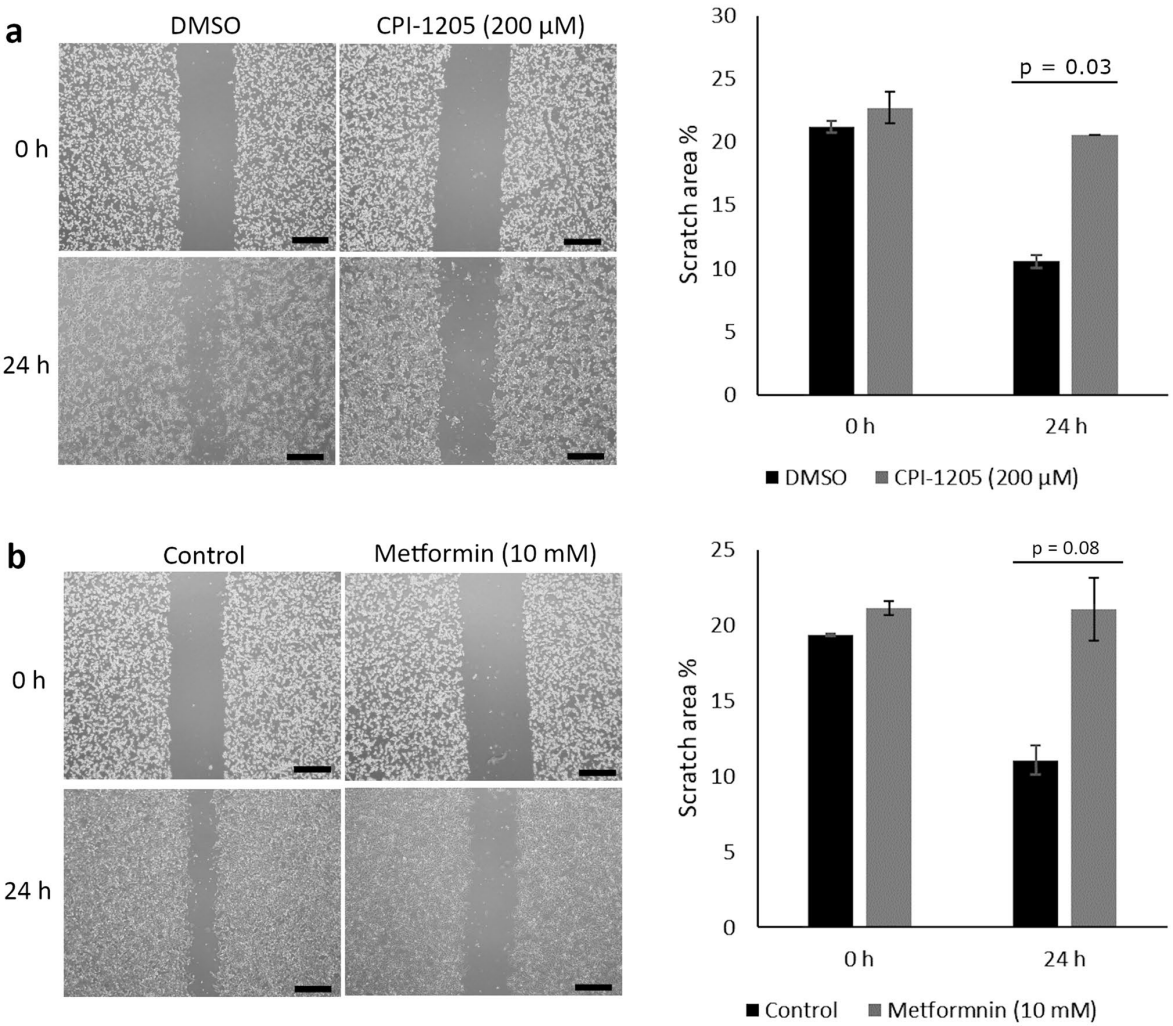
Effects of CPI-1205 and metformin on migration of SI-NET cells. Migration of CNDT2.5 cells was measured by scratch wound-healing assay following incubation with (**a**) 200 µM CPI-1205 or (**b**) 10 mM metformin. Representative bright-field images and quantification of scratch wound area at 0 and 24 h after treatment are shown. Scale bar 500 µm. Data presented are means ± SD of triplicate.

Scratch wound-healing assay was performed to investigate the effect of CPI-1205 and metformin on the migration of SI-NET cells in the presence of the cell proliferation inhibitor mitomycin C. We observed that the migration capacity of CNDT2.5 cells was significantly reduced (p = 0.03) after treatment with 200 µM CPI-1205 for 24 h, compared to the control (Fig. 7a). The effect of 10 mM metformin was not significant (p = 0.08) (Fig. 7b).

### The growth of GOT1 spheroids was arrested by CPI-1205 and metformin treatment

**Figure 8:**
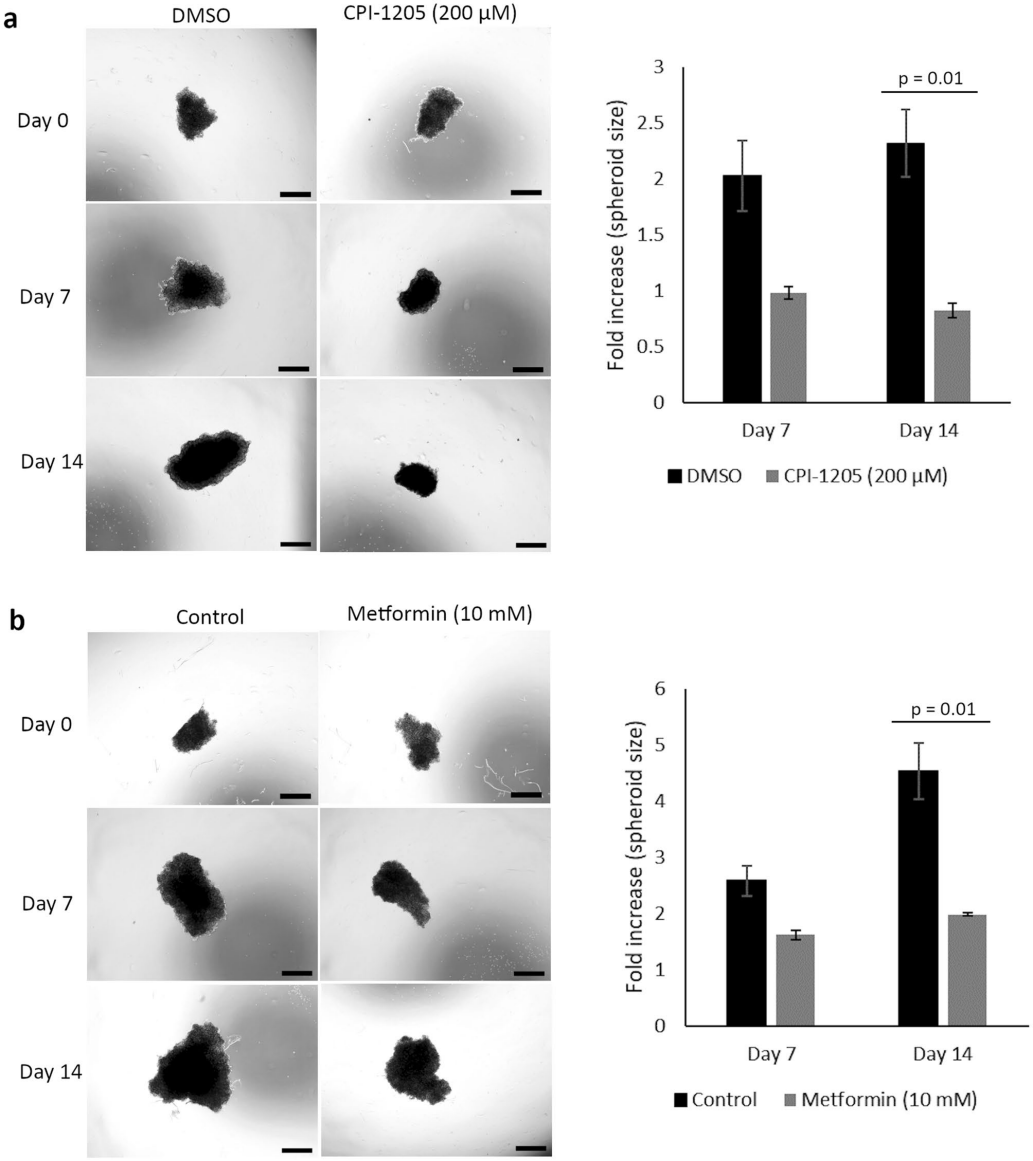
CPI-1205 and metformin treatment reduced the growth of GOT1 cell spheroids. Spheroids were treated with (**a**) 200 µM CPI-1205 or (**b**) 10 mM metformin for 14 days. Bright-field images were taken at day 0 of treatment, and 7 and 14 days after treatment. Scale bar 500 µm. The figure reports fold increases in spheroid size after 7 and 14 days of treatment compared to the controls. Data presented are means ± SD of triplicate.

Growing cancer cells in three-dimensional spheroid forms somewhat better resemble the in vivo microenvironment of tumors compared to monolayer cultures, and is an important tool to study the effect of different drugs and drug resistance^31^. Here, we induced GOT1 cells to form spheroids via self-assembly in a non-adhesive environment, and generated homogenous sized, single spheroids in multi-well plates to assess spheroid response to CPI-1205 or metformin treatment. The components were added to the culture medium 3 weeks after cell seeding when the spheroids had already formed. Bright-field images were obtained on the day of treatment (day 0), and day 7 and 14 after treatment. As shown in Fig. 8a, CPI-1205 treatment inhibited the growth and significantly reduced the size of spheroids compared to the control (p = 0.01). While the size of metformin treated spheroids increased slightly over 7 and 14 days, a higher fold increase in size was observed in the control spheroids (p = 0.01) (Fig. 8b), confirming that metformin treatment arrested the growth of spheroids.

## Discussion

Various studies have revealed oncogenic roles of EZH2 in a wide variety of cancers, and showed that EZH2 overexpression is positively correlated with increased tumor size, invasion, and poor clinical outcomes. Previously, we have shown that in parathyroid tumors, EZH2 is overexpressed and may have a crucial role in parathyroid tumorigenesis^32^. EZH2 is now considered as a potential target for cancer therapy, and different types of EZH2 inhibitors have been developed which are being evaluated in clinical trials. However, their potential to inhibit tumor growth in SI-NETs remains unexplored.

In this study, we investigated a possible oncogenic role of EZH2 in SI-NETs. We showed that EZH2 is strongly and diffusely expressed in all 38 tumors analyzed here using immunohistochemistry, whereas the immunofluorescence results demonstrated an undetectable expression of EZH2 in the enterochromaffin cells of the intestinal mucosa. As a consequence, we hypothesized that EZH2 activity may be required for SI-NET tumorigenesis. Indeed, accumulating studies in various types of cancer have highlighted that inhibition of EZH2 suppresses cancer initiation, progression, and metastases. In SI-NETs, we found that silencing EZH2 in vitro leads to a significant reduction in cell proliferation rate and induction of apoptosis. More importantly, EZH2 depletion suppressed the growth of SI-NET cells in the CNDT2.5 SI-NET xenograft mouse model. These findings support our hypothesis that EZH2 performs an important function in small intestinal neuroendocrine tumorigenesis.

In order to investigate whether inhibition of EZH2 may be of therapeutic benefit in patients with SI-NETs, we assessed the effect of several small molecule inhibitors of EZH2, including EPZ-6438 and CPI-1205, on the growth of SI-NET cell lines. The results showed that CPI-1205 could be a promising therapeutic drug for patients with SI-NETs. Previously, a significant antitumor activity of CPI-1205 was observed in a KARPAS-422 B-cell lymphoma xenograft model in mice^20^. Both CNDT2.5 and GOT1 cells used in this study express high levels of endogenous EZH2, and inhibition of EZH2 by CPI-1205 treatment repressed cell proliferation, promoted cell apoptosis, and reduced migration capacity of these cells. We also tested the EZH2 inhibitor GSK126 and found that the proliferation of CNDT2.5 cells was repressed.

Several meta-analyses and retrospective studies have reported a direct or indirect antitumor activity of metformin. In patients with advanced pancreatic NETs, retrospective studies confirmed a significant association between metformin treatment and longer progression-free survival^33,34^. However, further preclinical and prospective studies are now needed to demonstrate the effectiveness of metformin treatment when used in combination with standard treatments, and to investigate its mechanism of action. Metformin has been reported to decrease the level of H3K27me3 accompanied by EZH2 repression in ovarian cancer cells through activation of AMPK^28,35^. Activated AMPK has also been reported to disrupt the interaction between EZH2 and the suppressor of zeste 12 (SUZ12) by phosphorylating EZH2 in ovarian and breast cancer cells^36^. In the present study, metformin treatment reduced expression of EZH2, repressed cell viability in SI-NET cells, and showed a greater growth inhibitory effect in EZH2-expressing cells compared with EZH2-knockout cells. Therefore, in addition to the inhibitory effect of metformin on mTOR signaling pathway in NETs^37^, our results suggested that downregulation of EZH2 can also partially contribute to the metformin-antitumor effect in SI-NETs. However, no induction of apoptosis was detected upon metformin exposure. This could be because metformin has been proposed to either induce apoptosis or arrest cell cycle through AMPK-mediated p53 phosphorylation, depending on the cell specific p53-signaling and -mutational status^38^.

Furthermore, using a three-dimensional spheroid model of GOT1 cells, we demonstrated inhibitory effects of CPI-1205 and metformin on the growth of spheroids. We also tested the combination of CPI-1205 and metformin in both cell lines, and found that it could increase induction of apoptosis compared with mono-treatments, whereas cell proliferation was not affected. Considering the association of EZH2 and p53 pathway in NETs^39^, further research is warranted to investigate EZH2 association with the complex metformin signaling pathways in SI-NETs, and it will be of interest to investigate EZH2 downstream target genes in these tumors.

Taken together, our novel findings strongly suggest an important role for EZH2 as a candidate oncogene in SI-NETs that could be targeted for medical therapy in patients with SI-NETs. Our results also support the use of EZH2 specific inhibitor CPI-1205 and metformin as therapeutic options for these patients, alone or in combination with available medical treatments, including somatostatin analogues, peptide receptor radiotherapy, as well as mTOR inhibitor everolimus. However, future studies examining the effectiveness of these drugs in vivo are warranted, which may provide a strong rationale to initiate clinical trials in patients with SI-NETs.

## Methods

### Tissue specimens

In total 63 tumors and one small intestinal tissue specimen, from a total of 30 patients were included in this study (Supplementary Table S1). All tumors were obtained from patients diagnosed with SI-NET and operated upon in Uppsala University Hospital, and were handled according to institutional guidelines and regulations. Twenty-eight primary tumors, 27 mesenteric, 7 liver, 1 extra mesenteric lymph node metastases, and 1 normal small intestine tissue specimen were analyzed. Ethical approval from the Regional Ethical Review Board in Uppsala and informed written consent from the patients were achieved. Two human SI-NET cell lines were used in the experiments. CNDT2.5 adhesive cells^40,41^ developed from a liver metastasis from a patient diagnosed with primary ileal SI-NET, were kindly provided by Dr. Lee Ellis, MD, Anderson Cancer Center, Houston, TX, USA, and used in this study at cell passages 10–30. GOT1 adhesive cells^42,43^ were a kind gift from Dr. Ola Nilsson, Sahlgrenska Cancer Center, University of Gothenburg, Sweden. Both SI-NET cell lines expressed the neuroendocrine cell marker synaptophysin.

### Immunostaining

Paraffin-embedded tumor sections were deparaffinized with xylene and rehydrated through descending alcohol concentrations and distilled water. Background staining was blocked with 3% hydrogen peroxide. Then, the sections were heated in citrate buffer pH 6.0 and were treated with the proper normal serum and the rabbit monoclonal anti-EZH2 antibody (ab191080, Abcam), the rabbit monoclonal anti-synaptophysin antibody (ab32127, Abcam), the mouse monoclonal anti-Ki-67 antibody clone MIB-1 (M7240, Dako) or the rabbit polyclonal anti-caspase-3 active/cleaved form antibody (AB3623, Merck). After incubation with proper secondary antibody and Avidin–Biotin Complex, diaminobenzidine (DAB) was used for visualization. CNDT2.5 and GOT1 cells were fixed in formalin and incubated for 20 min with ice-cold 70% ethanol. Then the slides were stained for synaptophysin as described above. In order to estimate the proportion of positively stained cells for caspase-3 active form, a qualitative scoring system was performed; “++” was given for 50–95% of positive stained cells and “+” score for 10–49% of cells positive. Immunofluorescence: the sections were treated and incubated with a mouse monoclonal anti-chromogranin A antibody (LK2H10, Thermo Fisher Scientific) as mentioned above, followed by incubation with the proper fluorescence secondary antibody (Alexa 488, Life Technologies). Sections were washed three times with phosphate-buffered saline (PBS) containing 0.05% Tween20 and incubated with the anti-EZH2 antibody (ab191080, Abcam). Then, the sections were incubated with the fluorescence secondary antibody (Alexa 594, Life Technologies), washed again and mounted with Vectashield with DAPI (Vector Laboratories).

### RNA extraction and quantitative RT-PCR analysis

Frozen tumor sections were cut using a cryostat, and hematoxylin–eosin staining was used to select specimens with at least 80% tumor cells. Total RNA from the sections or the cells of SI-NET cell lines CNDT2.5 and GOT1 were isolated using RNeasy Plus Mini kit (Qiagen) according to the manufacturer’s instruction. DNase I treatment was performed on the extracted RNA using TURBO DNA-free kit (Life Technologies), followed by PCR analysis. Then, one microgram of the total RNA was converted to cDNA with the ‘First strand cDNA Synthesis kit’ (Thermo Fisher Scientific) using random hexamer primers, in accordance with the manufacturer’s instructions. The qRT-PCR reactions were run on StepOnePlus Real-Time PCR systems (Life Technologies) using TaqMan gene expression Master Mix and assays for EZH2 (Hs00544830_m1) and 18S rRNA (Hs03928990_g1) transcripts. EZH2 mRNA levels were normalized with the expression of the reference gene (18S rRNA). All samples were amplified in triplicate.

### Western blot analysis

Whole-cell protein lysates were extracted using Cytobuster Protein Extract Reagent (Merck) complemented with protease inhibitor cocktail (Roche). The membranes were blocked with 5% milk in PBS containing 0.1% Tween20 and then blotted with rabbit monoclonal anti-EZH2 antibody (ab191080, Abcam) or goat polyclonal anti-Actin (sc-1616, Santa Cruz Biotechnology). After washing, the membranes were incubated with the proper horseradish peroxidase-conjugated secondary antibodies. Finally, proteins were visualized using the enhanced chemiluminescence system (GE Healthcare).

### Cell transfection and drug treatment

CNDT2.5 cells were distributed onto six-well plates (10^6^) in DMEM-F12 complemented with 10% fetal bovine serum (Sigma Aldrich), 1% nonessential amino acids, and 1% penicillin–streptomycin (PEST), and were cultured at 37 °C in 5% CO_2_. After overnight incubation, the cells were transfected in triplicate with 4 μg EZH2 CRISPR double nickase plasmids (sc-417028-NIC h/h2, Santa Cruz Biotechnology) or control double nickase plasmid (sc-437281) using 8 μL of Lipofectamine 2000 transfection reagent (Life Technologies), according to the manufacturer’s instructions. Six hours after transfection, a fresh medium was added, complemented with 0.5 µg/mL puromycin (Invivogen).

GOT1 cells were cultured in RPMI-1640, complemented with 10% fetal bovine serum, 5 µg/mL insulin, and 5 µg/mL transferrin. CNDT2.5 (2 × 10^5^) and GOT1 (5 × 10^5^) cells were treated for 72 and 48 h, respectively, with several concentrations (50, 100, and 200 µM) of CPI-1205 (Selleckchem), an orally bioavailable selective inhibitor of EZH2, or with the vehicle, dimethyl sulfoxide (DMSO) (Sigma Aldrich), as the control. Both cell lines were treated with 2.5, 5, and 10 mM of metformin (sc-202000A, Santa Cruz Biotechnology) for 48 and 72 h, respectively, and distilled water was used as the vehicle. For the combination treatment, the two cell lines were incubated with CPI-1205 (200 µM) and metformin (10 mM) for 48 h. CNDT2.5 cells were treated with 1, 5, and 10 µM GSK126 (Selleckchem) or with the vehicle, DMSO, for 6 days. GSK126 stock solution in DMSO (10 mM) was incubated in 50 °C water bath for 30 min prior to use according to the manufacturer’s instructions.

### Cell proliferation assay and apoptosis

To assess cell proliferation, the CyQUANT cell proliferation assay kit (Invitrogen, Thermo Fisher Scientific) was used according to the manufacturer’s instructions. For each experiment, the same number of cells for all the samples was distributed in a 96-well plate and cell proliferation was followed for 24 h. After 24 h, the cells were frozen in the microplate and then lysed and stained with CyQuant GR dye solution. Infinite 200 PRO (TECAN) plate reader was used to measure fluorescence intensity at 480/520 nm. BrdU cell proliferation assay kit (Calbiochem) was used to measure proliferation in treated CNDT2.5 knockout cells, in CNDT2.5 and GOT1 cells treated with the combination treatment, and in CNDT2.5 cells treated with GSK126 according to the manufacturer’s instructions. BrdU was incubated with the cells for 24 h, and absorbance was measured at 450–540 nm using a plate reader. Apoptosis was measured using the Cell Death Detection ELISA kit (Roche), according to the manufacturer’s protocol. As a positive control, CNDT2.5 and GOT1 cells were incubated with 0.1 μg/mL camptothecin (Sigma Aldrich) for 48 h. Two or three biological replicates of each experiment were performed.

### Scratch wound-healing assay

CNDT2.5 cells were seeded into 6-well plates at a density of 10^6^ cells per well. Following overnight incubation, a scratch was introduced on the cell monolayer using a 100 μL pipette tip. Floating cells were washed away using PBS, and then cultured in media in triplicate with 200 μM CPI-1205 or 10 mM metformin. DMSO or distilled water in the media was used as controls. 6 μg/mL mitomycin C (Sigma Aldrich) was added to all wells to inhibit proliferation. The migration of cells into the scratch area was imaged at 0 and 24 h of the treatment using a light microscope (Invitrogen, Thermo Fisher Scientific). Then, the scratch wound areas were measured using the NIH Image-J software (1.53a) according to the program’s instructions. Two biological replicates of the experiments were performed.

### Formation and treatment of GOT1 spheroids

GOT1 cells were seeded in 24-well plates, coated with sterile agarose (Invitrogen, Thermo Fisher Scientific) gel (1%) to make a non-adhesive surface. The coated wells were incubated and washed three times with media before seeding the cells. 5 × 10^3^ cells were used to result in one spheroid per well. After 3 weeks of incubation, when a central necrosis area was formed^44^, the spheroids were treated with 200 μM CPI-1205 or 10 mM metformin in triplicate. DMSO or distilled water in the media was used as controls. The spheroids were photographed on the day of treatment (day 0), and at day 7 and 14 after treatment using a light microscope (Invitrogen, Thermo Fisher Scientific). The growth medium with additions was changed every 72 h. The size of spheroids was measured using the NIH Image-J software (1.53a) according to the program’s instructions. Fold increases were calculated by dividing the spheroid size at day 7 or day 14 by the spheroid size at day 0 for each individual spheroid. Two biological replicates of the experiment were performed.

### Animal model

The animal study was approved by the Uppsala Animal Ethics Committee (ID number 5.8.18-07081/2019). All experiments were performed according to the relevant regulations and the guidelines of ARRIVE (Animal Research: reporting of In Vivo Experiments). Female, NMRI-nude mice (5–6 weeks old) were purchased from Charles River. All mice were housed at the BMC animal facility (Uppsala University, Sweden) in individually ventilated cages (three mice per cage). Tumor implementation was performed 1 week after mouse delivery. CNDT2.5 cells (wild type, control-transfected or EZH2 knockout) were washed twice, counted, resuspended in PBS at 5 × 10^6^, and mixed 1:1 (vol/vol) with Matrigel (Corning) in a total volume of 100 µL. The cells were injected subcutaneously in the hind flank of the mice. Twice a week the mice were weighed, and tumor growth was monitored by caliper measurement. Tumor size was calculated using an ellipsoid volume formula (length × width × depth × π/6). Each treatment group included six mice. After 38 days, the mice were euthanized, and the xenograft tumors were dissected.

### Statistical analysis

Wilcoxon–Mann–Whitney *U* test was used to calculate differences in EZH2 mRNA expression between primary tumors and metastases. Student’s t-test was used to assess differences between two groups, and *p* values were adjusted using Bonferroni correction to compensate for multiple testing. One-way analysis of variance and Bonferroni correction were used for multiple comparisons between three biological groups. All data are presented as the mean ± standard deviation (SD), and p < 0.05 was considered to indicate a statistically significant difference. Statistics were calculated using R version 3.6.2 (2019-12-12).

## Supplementary Information

Supplementary Information.

## Data Availability

The datasets generated and/or analyzed during the present study are available from the corresponding authors on reasonable request.
