# Supplementary material for: EZH2 presents a therapeutic target for neuroendocrine tumors of the small intestine
Source: Sci Rep. 2021 Nov 23;11:22733. doi: 10.1038/s41598-021-02181-7 (PMC8611048; doi:10.1038/s41598-021-02181-7)
Supplement: Supplementary file 1 — Supplementary Information. [file 41598_2021_2181_MOESM1_ESM.pdf]

**EZH2 presents a therapeutic target for neuroendocrine tumors of the small intestine**

Elham Barazeghi*, Per Hellman, Olov Norlén, Gunnar Westin, and Peter Stålberg*

Department of Surgical Sciences, Uppsala University, Uppsala University Hospital, Rudbeck Laboratory, SE-751 85 Uppsala, Sweden.

per.hellman@surgsci.uu.se; olov.norlen@surgsci.uu.se; gunnar.westin@surgsci.uu.se

*Correspondence: elham.barazeghi@surgsci.uu.se and peter.stalberg@surgsci.uu.se

Supplementary Table S1: Clinical data for patients with SI-NETs

| Patients | Gender | Age at diagnosis | U-5-HIAA | CGA | ENETS grading | Survival (years) | Status |
|---|---|---|---|---|---|---|---|
| 1 | Male | 59 | 117 | 6.3 | 1 | 3.2 | AWD |
| 2 | Female | 63 | 28 | NA | 1 | 2.7 | DWD |
| 3 | Male | 59 | 165 | 47 | 1 | 2.3 | AWD |
| 4 | Female | 68 | NA | NA | 1 | 3.3 | AWOD |
| 5 | Male | 63 | 192 | NA | 2 | 4.3 | AWD |
| 6 | Male | 59 | 44 | 4.9 | 1 | 4.1 | AWOD |
| 7 | Female | 52 | 31 | 2.3 | 2 | 6.1 | AWD |
| 8 | Male | 53 | 92 | 9 | 1 | N/A | N/A |
| 9 | Female | 42 | 306 | NA | 1 | 7.8 | AWD |
| 10 | Male | 59 | 66 | 9.1 | 1 | 1.0 | AWD |
| 11 | Male | 51 | 87 | NA | 2 | 0.9 | DWD |
| 12 | Male | 74 | 32 | 3 | 1 | 0.3 | DWOD |
| 13 | Male | 72 | 104 | 7.4 | 1 | 3.0 | AWD |
| 14 | Male | 68 | 405 | NA | 1 | 3.5 | AWD |
| 15 | Male | 63 | 59 | 2.7 | 1 | 3.2 | AWD |
| 16 | Male | 79 | 84 | 18.3 | 2 | 5.1 | AWD |
| 17 | Female | 72 | NA | NA | 1 | 29.9 | DWD |
| 18 | Male | 64 | 41 | 4.6 | 1 | 2.8 | AWD |
| 19 | Male | 60 | 942 | NA | 1 | 3.3 | AWD |
| 20 | Male | 61 | 1564 | NA | 2 | 3.2 | AWD |
| 21 | Male | 61 | 47 | 4.8 | 1 | 1.3 | AWOD |
| 22 | Female | 73 | 33 | 3.9 | 2 | 0.6 | AWD |
| 23 | Male | 54 | 214 | 57 | 2 | 3.6 | DWD |
| 24 | Female | 76 | 59 | 11 | 1 | 7.8 | AWD |
| 25 | Male | 69 | <50 | 2.9 | 2 | 7.0 | AWOD |
| 26 | Female | 76 | 45 | 4.4 | 1 | 4.9 | AWD |
| 27 | Male | 68 | 20 | 4.9 | 2 | 10.1 | DWD |
| 28 | Female | 67 | 108 | 18 | 2 | 11.8 | AWD |
| 29 | Male | 67 | 697 | 26 | 1 | 11.4 | AWD |
| 30 | Female | 61 | 97 | 6.3 | 2 | 5.3 | AWD |

DWD dead with disease, DWOD dead without disease, AWD alive with disease, AWOD alive without disease

Supplementary Figure S1.

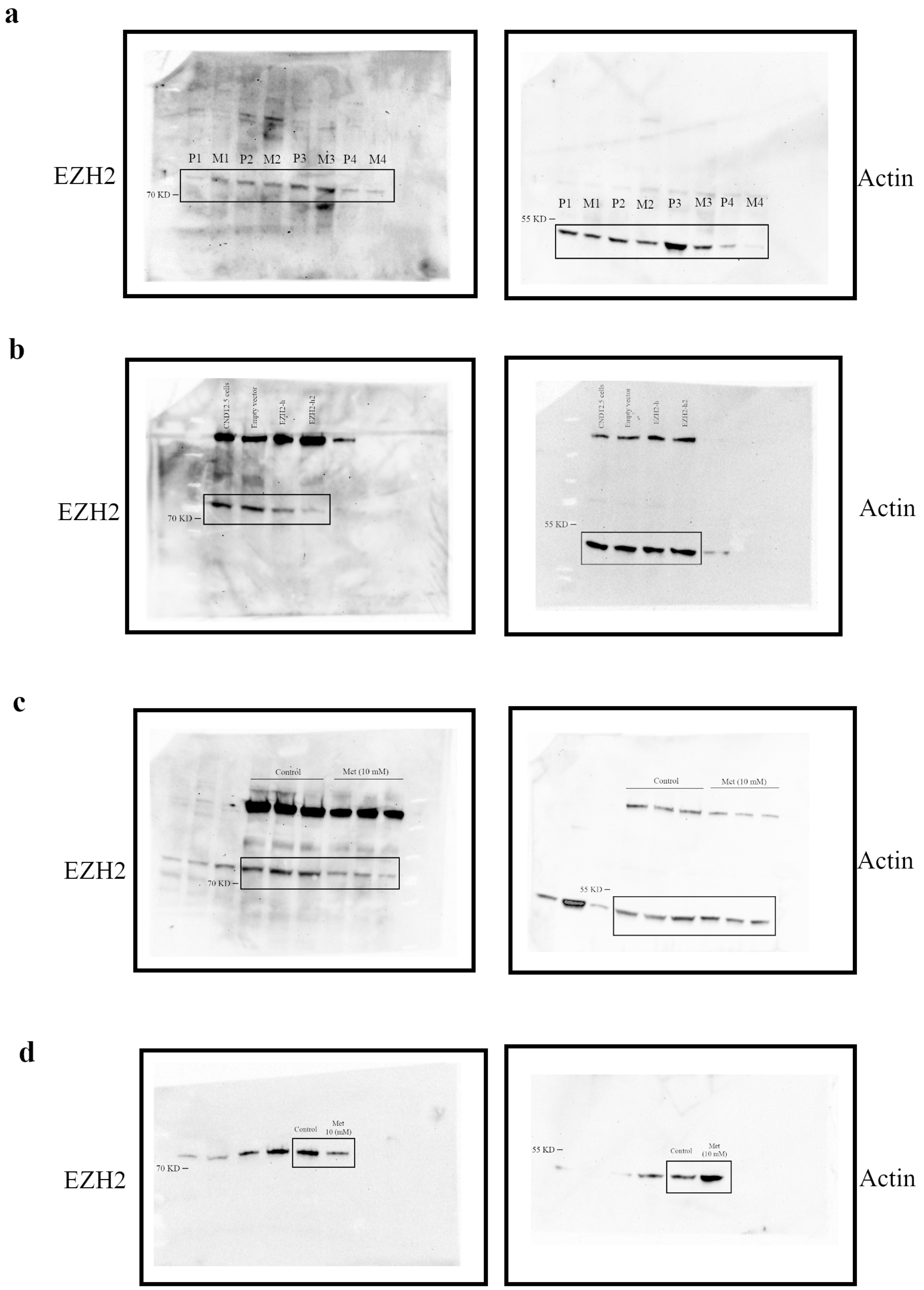


**Supplementary Figure S1.** Full-length blots for Figures (a) 1c, (b) 2a, (c) 5a, and (d) 5b in the manuscript. Actin was detected on the same membrane as loading control.

Supplementary Figure S2.

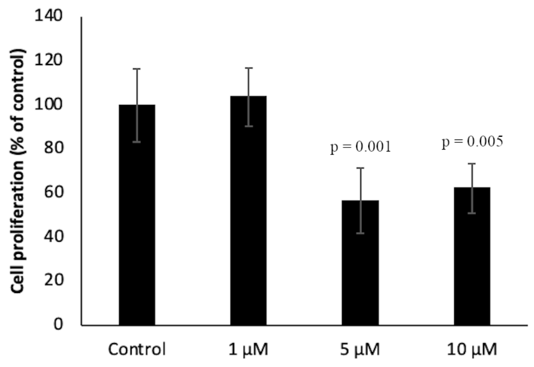


**Supplementary Figure S2.** GSK126 treatment in CNDT2.5 cells reduced proliferation compared with the control cells treated with DMSO. Cell proliferation was measured by BrdU assay after 6 days. Data shown are means ± S.D. of triplicate.

Supplemetary Figure S3.

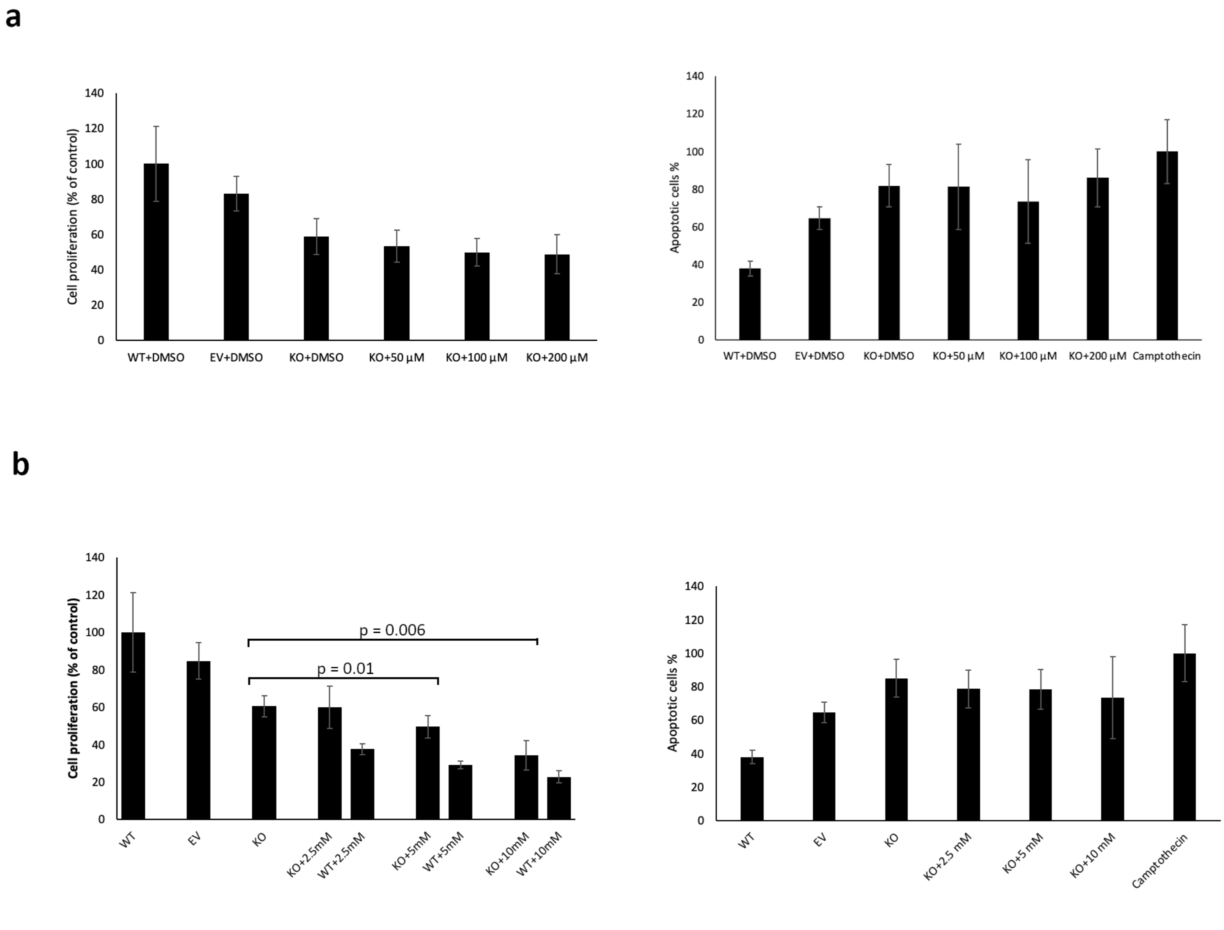


**Supplementary Figure S3.** CNDT2.5 EZH2 knockout cells (KO) treatment with (a) CPI-1205 and (b) metformin compared with the empty vector transfected cells (EV), and wildtype (WT). Cell proliferation was measured by BrdU assay, and apoptosis was analyzed by quantifying cytoplasmic histone-associated-DNA-fragments. Data shown are means ± S.D. of triplicate.

Supplementary Figure S4.

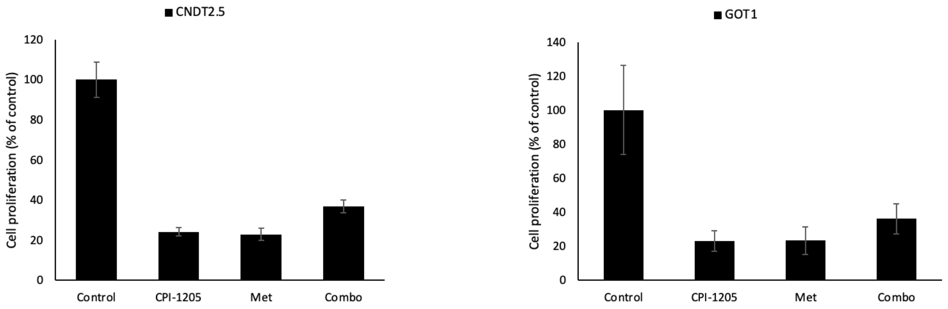


**Supplementary Figure S4**. CNDT2.5 and GOT1 cells were treated with CPI-1205 (200μM), metformin (Met) (10mM), or a combination of both (Combo), followed by BrdU proliferation assay. Data shown are means ± S.D. of triplicate.
